# Severe dengue in hospitalized adults at two tertiary referral hospitals in northern Vietnam: clinical features and outcomes

**DOI:** 10.1186/s41182-026-00999-3

**Published:** 2026-06-17

**Authors:** Nguyen Trong The, Vu Viet Sang, Pham Van Chung, Do Duc Anh, Tran Thi Thanh Huyen, Hoang Van Tong, Nguyen Linh Toan, Nguyen Minh Nam, Tomer Hertz, Le Huu Song, Thirumalaisamy P. Velavan

**Affiliations:** 1 108 Military Central Hospital, Hanoi, Vietnam; 2 Vietnamese-German Center for Medical Research (VG-CARE), Hanoi, Vietnam; 3 Institute of Tropical Medicine, University of Tübingen and German Center for Infection Research (DZIF), Tübingen, Germany; 4 Vietnam Military Medical University, Hanoi, Vietnam; 5 The Shraga Segal Department of Microbiology and Immunology, Ben-Gurion University of the Negev, Beer-Sheva, Israel; 6 Faculty of Medicine, Duy Tan University, Da Nang, Vietnam

**Keywords:** Severe dengue, Mortality, Shock, Metabolic dysfunction, Organ failure, Vietnam

## Abstract

**Background:**

Dengue remains a major global health concern; while most infections are self-limiting, a subset progresses to severe disease with substantial mortality. Updated data on severe dengue in hospitalized adults, particularly those managed in tertiary referral settings, remain limited. This study aimed to characterize the clinical features, laboratory abnormalities, and outcomes of adults with WHO-defined severe dengue admitted to two tertiary referral hospitals in northern Vietnam.

**Methods:**

We conducted a prospective observational cohort study of adults with WHO-defined severe dengue admitted to two tertiary referral hospitals in northern Vietnam between 2020 and 2024. Demographic, clinical, laboratory, and outcome data were systematically collected, and survivors were compared with non-survivors to identify factors associated with mortality.

**Results:**

A total of 104 adults with severe dengue were enrolled, of whom 31 (30%) died. Non-survivors were significantly older than survivors (median age 62 vs 45 years) and more frequently had hypertension (48% vs 23%; *p* = 0.011) and diabetes mellitus (45% vs 11%; *p* < 0.001). Shock occurred in 81% of non-survivors compared with 15% of survivors (*p* < 0.001). Fatal cases showed more frequent multi-organ failure and metabolic derangements, including acidosis with hyperlactatemia, renal dysfunction, hyperammonemia, coagulopathy, hypoalbuminemia, and elevated cardiac troponin I. In multivariable Firth logistic regression, shock (adjusted odds ratio [aOR] 8.59, 95% CI 2.38–36.27; *p* < 0.001) and cumulative organ dysfunction burden (aOR 2.69 per additional organ/system, 95% CI 1.57–5.36; *p* < 0.001) were independently associated with in-hospital mortality. Organ dysfunction burden also stratified time-to-event outcomes (log-rank *p* < 0.001).

**Conclusions:**

In this tertiary referral cohort of adults with WHO-defined severe dengue in northern Vietnam, mortality was high and was mainly associated with older age, comorbidities, shock, metabolic failure, and multi-organ dysfunction; shock plus cumulative organ dysfunction provided a practical risk-stratification signal.

## Background

Over the last two decades, there has been a marked increase in the incidence of dengue, driven by factors such as rapid urbanization, climate change, and expanding *Aedes* habitats. In 2024 alone, more than 14 million cases of dengue fever and approximately 9,500 deaths were reported on a global scale, reflecting the largest recorded annual burden to date [1]. Modelling studies estimate that about 390 million dengue infections occur annually, placing over 3.9 billion people at risk, with Asia bearing the greatest burden [2]. Beyond the large numbers observed in recent outbreaks, dengue severity spans a broad clinical spectrum, ranging from a self-limiting febrile illness to life-threatening disease.

The pathophysiology of severe dengue (SD) is multifactorial, involving a range of immune-mediated and endothelial-related factors, as well as increased vascular permeability and dysregulated inflammatory responses [3–5]. The 2009 classification of dengue by the World Health Organization (WHO) stipulates that severe dengue is characterized by the presence of severe plasma leakage, resulting in shock or respiratory distress, severe bleeding, or severe organ involvement including hepatic failure, encephalopathy, or myocardial dysfunction [6]. This classification continues to serve as the fundamental framework for clinical triage and management in endemic regions [7, 8]. Despite the advances in supportive care, the progression to severe dengue remains unpredictable and there is continuing mortality even in hospitalized patients. A substantial body of research has identified a correlation between advanced age and pre-existing comorbidities, with severe outcomes and mortality [9–11]. Concurrently, markers indicative of tissue hypoperfusion and metabolic failure, including hyperlactatemia and metabolic acidosis, have emerged as effective prognostic indicators, complementing traditional WHO warning signs [12–14].

Vietnam is a hyperendemic region for dengue, exhibiting significant annual fluctuations in case numbers and disease severity. In 2023 alone, approximately 369,000 cases of dengue fever were reported [15]. Although paediatric dengue has been the focus of extensive research in Southeast Asia, data concerning severe dengue in adults, particularly those requiring tertiary care, remain limited. This knowledge gap is becoming increasingly pertinent as dengue epidemiology shifts towards older age groups with chronic comorbidities, potentially altering clinical presentation, disease progression, and outcomes. Against this background, a prospective 5-year cohort study was conducted among hospitalized adults with severe dengue admitted to two tertiary referral centres in northern Vietnam. The primary objective was to comprehensively characterize the clinical manifestations, laboratory abnormalities, and outcomes of severe dengue in this cohort. Additionally, this study aimed to integrate readily available clinical parameters with detailed metabolic and organ-function markers to identify features that may facilitate early risk stratification and timely escalation of care in dengue-endemic settings where resources are limited.

## Methods

### Study design and ethical approval

We conducted a prospective observational cohort study between January 2020 and December 2024 at two tertiary referral hospitals in Hanoi, Vietnam: the Institute of Clinical Infectious Diseases, 108 Military Central Hospital, and Military Hospital 103. Both institutions serve as national referral centres for adult infectious diseases and participate in the Vietnam Dengue Surveillance Network.

The study protocol was approved by the Ministry of Science and Technology of Vietnam (Approval No. 11/2021/HĐ-NĐT) and by the institutional ethics committees of both participating hospitals (Ethics Approval No. 4763/HĐĐĐ). Prior to enrolment, written informed consent was obtained from all participants; for those under 18 years of age, consent was obtained from their parents or legal guardians. This study was conducted in accordance with Good Clinical Practice (GCP) and Good Clinical Laboratory Practice (GCLP) guidelines.

### Study population and patient recruitment

Patients aged ≥ 16 years admitted with laboratory-confirmed dengue infection were screened consecutively for eligibility. Dengue infection was confirmed by a positive NS1 antigen test and/or a positive anti-dengue IgM antibody test using a commercially available rapid immunochromatographic assay (SD Bioline Dengue Duo, Abbott, Korea). Patients were included if they fulfilled the WHO 2009 criteria for severe dengue, as adopted by the Vietnamese Ministry of Health [6]. Severe dengue was defined by the presence of at least one of the following criteria: severe plasma leakage resulting in dengue shock syndrome or respiratory distress; severe bleeding, including gastrointestinal or intracranial haemorrhage or bleeding requiring blood transfusion; or severe organ involvement, defined as hepatic injury (AST and/or ALT ≥ 1,000 U/L), acute kidney injury (serum creatinine ≥ 2 times baseline), significant neurological manifestations, or cardiac involvement. Exclusion criteria covered the presence of pre-existing end-stage organ failure, incomplete clinical or laboratory data at admission, or refusal to provide informed consent.

Demographic data, comorbidities, clinical features, complications, and outcomes were prospectively collected. The documented comorbidities included hypertension, diabetes mellitus, and other chronic medical conditions. The clinical complications that occurred during the patient’s hospitalization included shock, respiratory failure, acute kidney and liver failure, myocarditis, heart failure, and major bleeding. A range of laboratory tests were performed including haematological parameters, a coagulation profile, renal and liver function tests, serum lactate, ammonia, albumin, and cardiac biomarkers. All patients were managed according to institutional protocols for severe dengue aligned with Vietnamese Ministry of Health dengue diagnosis and treatment guidance and WHO recommendations. Management included close haemodynamic monitoring, fluid therapy, recognition and treatment of shock, and organ-supportive interventions when required.

The primary outcome of this study was in-hospital mortality. Patients were divided into two groups for the purposes of analysis: survivors and non-survivors. A comparison was made between the clinical and laboratory characteristics of these groups to identify factors associated with fatal outcomes.

### Statistical analysis

Data were analysed using R software version 4.3.2 (R Foundation for Statistical Computing, Vienna, Austria). The normality of continuous variables was assessed using the Shapiro–Wilk test, and the results are presented as the mean (standard deviation) or median [range], as appropriate. Categorical variables are expressed as absolute numbers and percentages. To draw comparisons between survivors and non-survivors, the appropriate statistical test was selected based on the distribution of the data. For categorical variables, the Chi-square test or Fisher's exact test was employed, while for continuous variables, the Welch's t-test or Wilcoxon rank-sum test was utilized. All tests were two sided, and a p-value of less than 0.05 was considered statistically significant.

Routine clinical and laboratory variables were assessed for mortality risk stratification. Categorical comparisons were reported with p-values, while multivariable Firth logistic regression provided adjusted odds ratios (ORs) and 95% confidence intervals (CIs). Analyses used available data only, with variable-specific denominators and no imputation; lactate models included only patients with lactate results.

Comorbidity, organ dysfunction, and major complication burdens were calculated by counting recorded conditions, affected organ/system domains, or major complications. Mortality was compared across burden categories of 0, 1–2, and ≥ 3 events, with trend testing using burden count as an ordinal/numeric predictor. Given 31 deaths among 104 patients, models were kept parsimonious. Predictors were selected a priori based on clinical relevance, univariable association, and avoidance of collinearity. Model performance was assessed using apparent AUC and repeated stratified five-fold cross-validation. Time-to-event analyses used hospital stay duration with Kaplan–Meier curves, log-rank tests, and Cox regression.

## Results

### Patient characteristics and outcomes

A total of 104 adults with WHO-defined severe dengue were enrolled during the study period. Overall, in-hospital mortality was 30% (31/104). All fatalities occurred in patients who met the WHO criteria for severe dengue, and these deaths were clinically attributable to severe dengue-associated complications, including shock, hyperlactatemia, metabolic acidosis, and organ failure. Baseline demographic and clinical characteristics stratified by survival status are summarized in Table 1. Non-survivors were significantly older than survivors (median age 62 vs 45 years; *p* = 0.007). Pre-existing comorbidities were substantially more common among non-survivors, particularly hypertension (48% vs 23%; *p* = 0.011) and diabetes mellitus (45% vs 11%; *p* < 0.001) (Table 1). Comorbidity burden showed a graded association with mortality: mortality was 11.4% among patients with no recorded comorbidity, 36.0% among those with one comorbidity, and 48.6% among those with two or more comorbidities (overall *p* = 0.001; trend *p* = 0.002).

**Table 1:** Demographic and comorbidities characteristics of severe dengue patients

	Non-survivors (*N* = 31)	Survivors (*N* = 73)	*P*-value
Days of illness at admission (days)	4 [2–7]	4 [2–7]	0.514^!^
Sex (male)	14 (45%)	39 (53%)	0.441^$^
Age (years)	62 [25–92]	45 [16–86]	**0.007**^**!**^
Comorbidities			
Hypertension	15 (48%)	17 (23%)	**0.011**^$^
Diabetes	14 (45%)	8 (11%)	** < 0.001**^$^
Gastritis/peptic disease	2 (7%)	9 (12%)	0.499^*^
Alcoholic liver disease	2 (7%)	3 (4%)	–
Cancer	3 (10%)	1 (1%)	–
Hepatitis B	2 (7%)	0	–
COPD	2 (7%)	1 (1%)	–
Chronic kidney disease	3 (10%)	3 (4%)	–
Heart failure history	2 (7%)	1 (1%)	–
No comorbidities	7 (23%)	41 (56%)	**0.002**^$^

Variables are summarized as median [range] for continuous data with non-normal distributions or absolute counts (percentages) for categorical data. *P*-values were determined from comparisons between non-survivors (*n* = 31) and survivors (*n* = 73), using the Chi-square test ($), Fisher test (*), or Wilcoxon rank-sum test (!). *P*-values in bold indicate statistical significance. *COPD* Chronic Obstructive Pulmonary Disease

### Clinical manifestations and complications

Initial clinical symptoms were largely comparable between survivors and non-survivors (Table 2). Abdominal pain was reported more frequently in survivors than in non-survivors (53% vs 29%; *p* = 0.022), whereas no significant differences were observed for rash, vomiting, fluid accumulation, or bleeding manifestations at presentation. Marked differences emerged in the occurrence of major complications during hospitalization. Shock developed in 81% of non-survivors compared with 15% of survivors (*p* < 0.001) (Table 2). Similarly, non-survivors exhibited substantially higher rates of multi-organ failure, including respiratory failure (97% vs 21%; *p* < 0.001), acute kidney failure (71% vs 10%; *p* < 0.001), acute liver failure (32% vs 7%; *p* = 0.002), myocarditis (45% vs 14%; *p* < 0.001), and heart failure (23% vs 4%; *p* = 0.007) (Table 2). These findings indicate that fatal outcomes were strongly associated with shock and widespread organ dysfunction rather than isolated clinical manifestations.

**Table 2:** Clinical manifestations of severe dengue patients at admission

	Non-survivors (*N* = 31)	Survivors (*N* = 73)	*P*-value
Symptoms			
Rash	6 (19%)	13 (18%)	0.852^!^
Abdominal pain	9 (29%)	39 (53%)	**0.022**^!^
Frequent vomiting	8 (26%)	25 (34%)	0.398^!^
Fluid accumulation	18 (58%)	29 (40%)	0.086^!^
Bleeding manifestations			
Mucosal bleeding	10 (32%)	32 (44%)	0.271^!^
Severe bleeding	10 (32%)	11 (15%)	0.046^!^
No bleeding	11 (34%)	30 (41%)	0.592^!^
Major complications			
Shock	25 (81%)	11 (15%)	** < 0.001**^!^
Severe liver injury	16 (52%)	47 (64%)	0.223^!^
Respiratory failure	30 (97%)	15 (21%)	< 0.001^*^
Kidney failure	22 (71%)	7 (10%)	** < 0.001**^!^
Liver failure	10 (32%)	5 (7%)	0.002^*^
Myocarditis	14 (45%)	10 (14%)	** < 0.001**^!^
Heart failure	7 (23%)	3 (4%)	0.007^*^

Variables are summarized as absolute counts (percentages) for categorical data. *P*-values were determined from comparisons between non-survivors (*n* = 31) and survivors (*n* = 73), using the Chi-square test (!) or Fisher test (*). *P*-values in bold indicate statistical significance

When complications were analysed as cumulative burden rather than as mutually exclusive events, mortality increased from 0% in patients without organ dysfunction to 37.5% in those with 1–2 organ/system dysfunctions and 88.9% in those with ≥ 3 organ/system dysfunctions (overall *p* < 0.001; trend *p* < 0.001). A similar gradient was observed for major complication burden, with mortality rates of 0%, 22.5%, and 78.6% in patients with 0, 1–2, and ≥ 3 major complications, respectively (overall *p* < 0.001; trend *p* < 0.001) (Table 3).

**Table 3:** Mortality according to cumulative comorbidity, organ dysfunction, and major complication burden

Burden	Category	*N*	Deaths	Survivors	Mortality (%)	*p*-value overall	*p*-value trend
Comorbidity burden	0	44	5	39	11.4	0.001	0.002
Comorbidity burden	1	25	9	16	36.0	0.001	0.002
Comorbidity burden	≥ 2	35	17	18	48.6	0.001	0.002
Organ dysfunction burden	0	46	0	46	0.0	< 0.001	< 0.001
Organ dysfunction burden	1–2	40	15	25	37.5	< 0.001	< 0.001
Organ dysfunction burden	≥ 3	18	16	2	88.9	< 0.001	< 0.001
Major complication burden	0	36	0	36	0.0	< 0.001	< 0.001
Major complication burden	1–2	40	9	31	22.5	< 0.001	< 0.001
Major complication burden	≥ 3	28	22	6	78.6	< 0.001	< 0.001

Variables are summarized as absolute counts and percentages. Overall *p*-values compare mortality across burden categories, and trend *p*-values evaluate increasing burden as an ordinal/numeric predictor. Organ dysfunction burden includes respiratory, renal, hepatic, myocardial, and cardiac failure. Major complication burden includes shock, severe bleeding, respiratory failure, renal failure, liver failure, myocarditis, and heart failure

### Laboratory findings

Laboratory abnormalities differed markedly between survivors and non-survivors (Table 4). Non-survivors demonstrated significantly lower haemoglobin and haematocrit levels and higher white blood cell counts, consistent with relative anaemia and leucocytosis. Metabolic derangements were prominent among non-survivors. They exhibited more severe metabolic acidosis, reflected by a lower mean arterial pH (7.23 vs 7.46; *p* < 0.001), and significantly higher serum lactate concentrations (5.50 vs 2.35 mmol/L; *p* = 0.003). Indicators of end-organ dysfunction were also more pronounced in non-survivors, including higher serum creatinine (158.8 vs 87.4 µmol/L; *p* < 0.001) and ammonia levels (163.7 vs 75.7 µmol/L; *p* = 0.002) (Table 4). Evidence of hepatic synthetic dysfunction and plasma leakage was reflected by higher international normalized ratio (INR; 1.31 vs 1.11; *p* = 0.019) and lower serum albumin levels (28.7 vs 32.0 g/L; *p* = 0.026) in non-survivors (Table 4). Additionally, non-survivors showed significantly elevated cardiac troponin I levels (Troponin; 281.11 vs 34.4 ng/L; *p* = 0.001), indicating a higher burden of myocardial injury. Overall, fatal severe dengue was characterized by profound metabolic acidosis, tissue hypoperfusion, and multi-organ failure, with laboratory abnormalities closely paralleling the observed clinical severity.

**Table 4:** Laboratory findings of severe dengue patients at admission

	Non-survivors (*N* = 31)	Survivors (*N* = 73)	*P*-value
Haematology			
Haemoglobin (g/dL)	11.3 [5.5–17.4]	14.9 [4.8–19.2]	0.002^*^
RBC count (× 10^12^/L)	3.90 (1.34)	4.81 (1.07)	**0.004**^**#**^
Haematocrit	0.34 [0.16–0.52]	0.43 [0.15–0.54]	0.007^*^
WBC count (× 10⁹/L)	8.34 [1.2–23.1]	5.38 [1.52–19.2]	0.016^*^
Platelet count (× 10⁹/L)	21.5 [7–92]	24.5 [3–128]	0.983^*^
Sepsis/Inflammation			
Troponin (ng/L)	281.11 [6.83–13699.1]	34.4 [4.28–14764.6]	0.001^*^
Procalcitonin (ng/mL)	2.1 [0.34–465]	1.61 [0.32–32.69]	0.361^*^
CRP (mg/L)	51.35 [0.61–257.88]	24.88 [0.76–164.59]	0.053^*^
Blood gas and perfusion			
Arterial pH (unitless)	7.23 (0.2)	7.46 (0.07)	** < 0.001**^**#**^
Blood lactate (mmol/L)	5.50 [0.5–21.88]	2.35 [1.2–12.2]	0.003^*^
pCO₂ (mmHg)	32 [12.5–69]	31.6 [13–110]	0.842^*^
pO₂ (mmHg)	88 [25–280]	95 [22–172]	0.977^*^
Ammonia (µmol/L)	163.7 [33.9–690.7]	75.7 [45.2–136.9]	0.002^*^
Renal electrolytes			
Creatinine (µmol/L)	158.81 [45–465.68]	87.39 [54.6–429.15]	< 0.001^*^
Sodium (mmol/L)	138.57 (11.86)	132.84 (4.61)	**0.019**^**#**^
Coagulation			
INR	1.31 [0.86–3.79]	1.11 [0.91–3.2]	0.019^*^
Fibrinogen (g/L)	1.94 (1.28)	2.54 (1.01)	0.076^#^
Liver function			
AST (U/L)	655.2 [58.2–25997.8]	1274.3 [54.3–9726.8]	0.367^*^
ALT (U/L)	378.9 [22–6089.7]	640.4 [27.6–3672.6]	0.949^*^
Total bilirubin (µmol/L)	22.8 [3.8–106.1]	16.15 [5.3–287.8]	0.136^*^
Direct bilirubin (µmol/L)	9.5 [0.67–61.03]	5 [1.32–171.01]	0.065^*^
Albumin (g/L)	28.68 (7.04)	31.98 (5.08)	**0.026**^**#**^

Variables are summarized as median [range] for continuous data with non-normal distributions or mean [SD] for those with normal distributions. *P*-values were determined from comparisons between non-survivors [*n* = 31] and survivors [*n* = 73], using the Welch’s t-test [#] or Wilcoxon rank-sum test [*]. *P*-values in bold indicate statistical significance. *RBC* Red blood cell count, *WBC* White blood cell count, *PLT* Platelet count, *PCT* Procalcitonin, *pCO₂* arterial partial pressure of carbon dioxide, *pO₂* arterial partial pressure of oxygen, *INR* International Normalized Ratio, *AST* Aspartate aminotransferase, *ALT* Alanine aminotransferase, *CRP* C-reactive protein

### Multivariable mortality models and internal validation

In the primary clinical/organ dysfunction model including age, comorbidity burden, shock, and organ dysfunction burden, shock and cumulative organ dysfunction burden remained independently associated with in-hospital mortality. Shock was associated with an aOR of 8.59 (95% CI 2.38–36.27; *p* < 0.001), and each additional organ/system dysfunction was associated with an aOR of 2.69 (95% CI 1.57–5.36; *p* < 0.001) (Table 5).

**Table 5:** Multivariable mortality models

Model	Variable	*N*	Events	Adjusted OR (95% CI)	*p*
A: Clinical/organ dysfunction burden model	Age, per 10 years	104	31	1.42 (0.95–2.27)	0.093
A: Clinical/organ dysfunction burden model	Comorbidity burden, per additional condition	104	31	1.37 (0.85–2.36)	0.200
A: Clinical/organ dysfunction burden model	Shock	104	31	8.59 (2.38–36.27)	< 0.001
A: Clinical/organ dysfunction burden model	Organ dysfunction burden, per additional organ/system	104	31	2.69 (1.57–5.36)	< 0.001
B: Clinical + lactate model	Age, per 10 years	50	28	1.51 (0.96–2.56)	0.077
B: Clinical + lactate model	Comorbidity burden, per additional condition	50	28	1.22 (0.66–2.29)	0.510
B: Clinical + lactate model	Shock	50	28	11.54 (2.59–72.22)	< 0.001
B: Clinical + lactate model	Blood lactate, per 2 mmol/L	50	28	1.34 (0.99–1.94)	0.055
C: Major complication burden model	Age, per 10 years	104	31	1.56 (1.02–2.64)	0.041
C: Major complication burden model	Comorbidity burden, per additional condition	104	31	1.38 (0.86–2.40)	0.184
C: Major complication burden model	Major complication burden, per additional complication	104	31	4.11 (2.42–9.15)	< 0.001

Adjusted ORs and 95% CIs were estimated using Firth penalized logistic regression. Age was modelled per 10-year increase; comorbidity burden per additional condition; organ dysfunction burden per additional organ/system; blood lactate per 2 mmol/L increase; and major complication burden per additional complication. Model B was restricted to patients with available lactate measurements. *OR* odds ratio, *CI* confidence interval

Age and comorbidity burden were associated with mortality in univariable and burden analyses but were attenuated after adjustment for shock and organ dysfunction. The apparent AUC of this model was 0.942, and the repeated stratified five-fold cross-validated AUC was 0.921. In a prespecified clinical-plus-lactate model restricted to patients with available lactate measurements, shock remained independently associated with mortality, while lactate showed a borderline adjusted association (aOR 1.34 per 2 mmol/L increase, 95% CI 0.99–1.94; *p* = 0.055) (Table 5).

### Time-to-event analysis

**Fig. 1:**
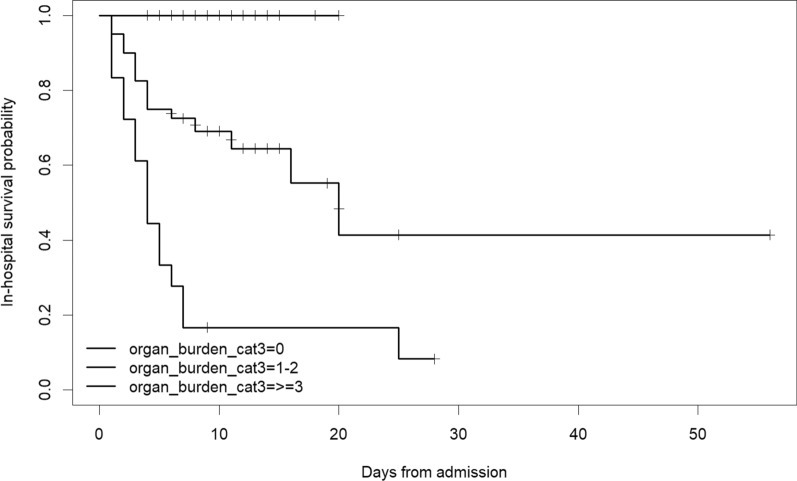
Kaplan–Meier in-hospital survival curves stratified by organ dysfunction burden category. In-hospital survival probability was compared across three groups according to the number of organ/system dysfunctions: 0**,** 1–2, and ≥ 3. Patients with a higher organ dysfunction burden had a markedly lower probability of survival during hospitalization. Tick marks indicate censored observations. The difference between groups was statistically significant (log-rank *p* < 0.001)

Kaplan–Meier analysis showed significant separation of in-hospital survival curves by organ dysfunction burden category (log-rank *p* < 0.001) (Fig. 1). In Cox proportional hazards regression adjusted for age, comorbidity burden, shock, and organ dysfunction burden, shock remained associated with mortality over time (HR 6.59, 95% CI 2.43–17.84; *p* < 0.001), and each additional organ/system dysfunction was associated with an increased hazard of death (HR 1.36, 95% CI 1.08–1.71; *p* = 0.010) (Fig. 1).

## Discussion

Dengue remains a significant public health threat in Vietnam [16, 17]. While most infections are self-limiting, a subset progresses to severe disease, which is associated with substantial mortality. Our study reaffirms that fatal outcomes were associated with advanced age, pre-existing comorbidities, shock, and multi-organ failure.

The population-level mortality rate from dengue in Vietnam is relatively low. According to the World Health Organization (WHO) office in Vietnam, dengue mortality has remained consistently low since 2005, at fewer than one death per 1,000 reported cases [18]. In this cohort, the observed mortality rate was around 30%. Although this is relatively high compared with many hospital-based reports on dengue from endemic areas [19, 20], it is consistent with mortality estimates reported for adults with severe dengue requiring intensive care or referral to a specialist hospital, including 25% among adults with severe dengue admitted to intensive care units on Réunion Island [21] and 31% in a multicentre study of patients with severe dengue in Taiwan [22]. These variations likely reflect differences in case definitions, age distribution, burden of comorbidities, referral patterns, time of presentation, access to intensive supportive care, and the proportion of patients with shock or multi-organ failure. It is important to note that our cohort was limited to adults with WHO-defined severe dengue admitted to tertiary referral hospitals and should not be interpreted as representative of the overall dengue case-fatality rate in Vietnam.

This cohort comprised adults with WHO-defined severe dengue admitted to tertiary referral hospitals, many of whom had shock, comorbidities, metabolic derangements, and multi-organ dysfunction. Fatal cases were therefore characterized by shock, metabolic failure, and multiple organ failure, and frequently occurred in older patients with underlying conditions. Advanced age and chronic comorbidities were important unadjusted determinants of mortality: non-survivors were older and more likely to have hypertension and diabetes than survivors, consistent with prior evidence linking ageing and cardiometabolic disease to worse dengue outcomes [23]. Patients of advanced age may have diminished physiological reserve and augmented endothelial dysfunction, rendering them more susceptible to severe plasma leakage, shock, and organ injury. As the epidemiology of dengue shifts increasingly towards adult and elderly populations in Asia, these factors are likely to play an expanding role in disease severity and mortality [6]. These findings emphasize the impact of host factors on dengue progression and support enhanced clinical surveillance and prompt intervention in older adults and patients with chronic disease [24].

Shock and multi-organ failure emerged as the most significant clinical discriminators distinguishing survivors from non-survivors. In infectious diseases, shock is a recognized final common pathway leading to death, and septic shock is associated with in-hospital mortality rates exceeding 40% [25]. In dengue haemorrhagic fever/dengue shock syndrome, once shock develops, reported fatality has ranged from 12 to 44%, although outcomes improve substantially in centres providing appropriate intensive supportive care [26]. In addition, our data showed that nearly all non-survivors progressed to respiratory, renal, hepatic, and/or cardiac failure. This pattern underscores the notion that fatal dengue is typically driven by systemic circulatory collapse and organ dysfunction rather than isolated complications [6]. Although severe bleeding was more frequent among non-survivors than survivors (32% vs 15%; *p* = 0.046), the overall mortality pattern was more strongly characterized by shock, metabolic failure, and organ dysfunction than by haemorrhage alone. This observation lends support to a shift in clinical focus towards the early recognition and aggressive management of shock and organ dysfunction, as opposed to an exclusive emphasis on bleeding manifestations [10]. The multivariable analyses strengthen this interpretation: after adjustment for age and comorbidity burden, shock and cumulative organ dysfunction burden remained independently associated with mortality. This finding indicates that the risk signal in this cohort was not driven by any single isolated complication, but by the combined burden of circulatory collapse and progressive organ-system involvement.

The laboratory findings closely reflected the clinical severity observed in non-survivors. Tissue hypoperfusion and metabolic collapse, indicated by higher lactate levels and metabolic acidosis, were more pronounced in patients who died. Elevated lactate levels are likely to reflect circulatory failure and impaired oxygen delivery [23]. In this cohort, non-survivors had lower arterial pH and approximately two-fold higher median blood lactate levels than survivors. These findings are consistent with previous studies suggesting that lactate may help identify patients at risk of shock or clinical deterioration in severe dengue [12, 13]. However, in the clinical-plus-lactate model, lactate showed only a borderline adjusted association with mortality after accounting for shock. Therefore, lactate should be interpreted as a supportive marker of tissue hypoperfusion and metabolic failure rather than as an independent prognostic marker.

Acute kidney injury has consistently been associated with increased mortality in dengue fever [23], while liver failure may result in hyperammonaemia. Hepatic dysfunction with hyperammonaemia is less consistently emphasized in the dengue literature but is biologically plausible as a contributor to encephalopathy in severe cases. In non-survivors, there was also a marked incidence of renal and potential hepatic dysfunction, as indicated by elevated creatinine and ammonia levels, in addition to coagulopathy and hypoalbuminemia. Non-survivors had significantly lower serum albumin levels, reflecting more pronounced plasma leakage and/or impaired hepatic synthetic function, along with higher international normalized ratio, consistent with coagulopathy. In addition, evidence of myocardial involvement was prominent: median cardiac troponin I levels were approximately eight-fold higher in non-survivors and were associated with a markedly higher incidence of myocarditis (45% vs 14% in survivors). Although myocarditis is recognized within the WHO criteria for severe dengue, our findings suggest that elevated cardiac troponin I may reflect greater myocardial injury and more severe systemic disease among fatal cases [27, 28].

A key implication of the revised analysis is that cumulative burden metrics may be more informative for bedside triage than long lists of individual complications. The organ dysfunction burden model retained all 104 patients, limited the number of correlated organ-failure predictors included in the model, and showed good internal discrimination. These findings suggest that shock combined with increasing organ/system dysfunction may help identify patients who require closer monitoring and escalation of supportive care in tertiary referral settings. However, external validation is needed before these findings can be used as a routine clinical risk-stratification approach.

Limitations of this study should be acknowledged. First, the cohort was derived from two tertiary referral hospitals, which may limit generalizability and may partly explain the high observed mortality through referral bias and delayed presentation or referral. Secondly, the study exclusively included adult patients, and therefore, the findings may not be applicable to paediatric populations, where dengue pathophysiology and outcomes differ [9]. Thirdly, a systematic assessment of virological factors such as infecting serotype, viral load, and immune status (primary versus secondary infection) was lacking, thereby preventing their evaluation as predictors of severity. In addition, the analysis used the most abnormal laboratory parameters recorded during hospitalization; serial measurements could provide additional insight into disease trajectory and prognosis. Additional limitations relate to the revised modelling strategy. The number of fatal events was modest; therefore, adjusted models were intentionally parsimonious and Firth penalized logistic regression was used to reduce small-sample and separation bias. The reported AUCs should be interpreted as internal validation rather than external validation, and the findings require confirmation in independent cohorts. Time-to-event analyses used hospital stay from admission to death or discharge as the available time scale and should be interpreted in that context. Finally, although admission timing and routinely available dengue diagnostic markers were considered, these markers cannot replace systematic serotyping, viral-load measurement, genotyping, or definitive classification of primary versus secondary infection.

## Conclusions

In this cohort of adults with WHO-defined severe dengue admitted to two tertiary referral hospitals in northern Vietnam, severe dengue was associated with substantial in-hospital mortality, particularly among older patients and those with comorbidities. Fatal outcomes were mainly linked to shock, metabolic derangement, and multi-organ dysfunction. After adjustment, shock and cumulative organ dysfunction burden were the strongest mortality signals, and organ dysfunction burden also stratified in-hospital survival. These findings suggest that cumulative organ dysfunction assessment, together with routine metabolic and organ-function markers, may help identify patients requiring urgent escalation of supportive care, but external validation in independent cohorts is warranted.

## Data Availability

This study generated and analysed original clinical and laboratory research data from a prospective observational cohort.
